# Evidence for rapid weathering response to climatic warming during the Toarcian Oceanic Anoxic Event

**DOI:** 10.1038/s41598-017-05307-y

**Published:** 2017-07-10

**Authors:** Theodore R. Them, Benjamin C. Gill, David Selby, Darren R. Gröcke, Richard M. Friedman, Jeremy D. Owens

**Affiliations:** 1 Department of Geosciences, Virginia Polytechnic Institute and State University, Blacksburg, Virginia 24061 USA; 2 Department of Earth, Ocean and Atmospheric Science & National High Magnetic Field Laboratory, Florida State University, Tallahassee, Florida 32306 USA; 3 Department of Earth Sciences, Durham University, Durham, DH1 3LE UK; 4 Pacific Centre for Isotopic and Geochemical Research, Department of Earth, Ocean and Atmospheric Sciences, University of British Columbia, Vancouver, V6T 1Z4 Canada

## Abstract

Chemical weathering consumes atmospheric carbon dioxide through the breakdown of silicate minerals and is thought to stabilize Earth’s long-term climate. However, the potential influence of silicate weathering on atmospheric *p*CO_2_ levels on geologically short timescales (10^3^–10^5^ years) remains poorly constrained. Here we focus on the record of a transient interval of severe climatic warming across the Toarcian Oceanic Anoxic Event or T-OAE from an open ocean sedimentary succession from western North America. Paired osmium isotope data and numerical modelling results suggest that weathering rates may have increased by 215% and potentially up to 530% compared to the pre-event baseline, which would have resulted in the sequestration of significant amounts of atmospheric CO_2_. This process would have also led to increased delivery of nutrients to the oceans and lakes stimulating bioproductivity and leading to the subsequent development of shallow-water anoxia, the hallmark of the T-OAE. This enhanced bioproductivity and anoxia would have resulted in elevated rates of organic matter burial that would have acted as an additional negative feedback on atmospheric *p*CO_2_ levels. Therefore, the enhanced weathering modulated by initially increased *p*CO_2_ levels would have operated as both a direct and indirect negative feedback to end the T-OAE.

## Introduction

The chemical weathering of rocks constitutes a negative and stabilizing feedback to Earth’s long-term (10^8^–10^9^ yr) climate by consuming atmospheric CO_2_, modulating the greenhouse effect and, in turn, global temperatures^1–3^. On these timescales, chemical weathering is dominantly regulated by tectonics, atmospheric *p*CO_2_, temperature, the lithology of materials being weathered, and the strength of the hydrological cycle^3^. Although the influence of weathering on long-term climate is well established^3^, much less is known about how this process potentially operates and influences climate on shorter times scales (<10^6^ yr)^4^.

**Figure 1:**
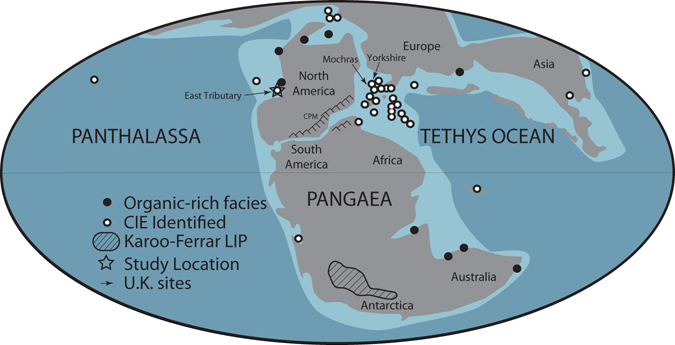
Global palaeogeography of the Early Toarcian (modified from ref. 71). Star represents this study’s location. Arrows point to the UK study locations^20, 23^, which are geographically close to one another. Hatched outline in southern Pangaea (present-day southern Africa and Antarctica) represents location and known extent of Karoo-Ferrar Large Igneous Province. Dark grey represents landmasses, light blue represents shallow seas, and dark blue represents open oceans. CPM = Central Pangaean Mountains. See ref. 15 for a list of locations that document the T-OAE CIE.

The T-OAE of the Early Jurassic Period constituted an ephemeral interval of global warming, perturbations in the global carbon cycle^5^, widespread oceanic anoxia^6^, and elevated marine extinction rates^7^. These environmental and ecological changes have been linked to the emplacement of the Karoo-Ferrar Large Igneous Province (LIP) and subsequent injection of greenhouse gases into the atmosphere^8^ (Fig. 1). Specifically, the addition of mantle-derived CO_2_ and thermogenic CH_4_ derived from the emplacement of the LIP^9–11^ and subsequent releases of CH_4_ from marine clathrates^12, 13^ and terrestrial environments^14, 15^ to the oceans and atmosphere are the proposed drivers of the T-OAE warming and carbon cycle perturbations. These perturbations are now recorded in sedimentary successions as pronounced negative carbon isotope excursions (CIEs), which occurred during a long-term trend to more positive carbon isotope values. This negative excursion is followed by a positive CIE thought to be the result of enhanced organic matter burial under anoxic conditions in marine and lacustrine environments^5, 6^. Collectively, these two carbon isotope excursions are used to stratigraphically define the T-OAE interval.

Under the enhanced greenhouse effect triggered by elevated levels of atmospheric greenhouse gases during the T-OAE, global temperatures would have increased and the hydrological cycle would have strengthened^5^. Rising *p*CO_2_, global temperatures, and precipitation rates would have led to accelerated weathering rates^3^. To investigate the proposition of accelerated weathering during the T-OAE, we have utilized osmium isotope (^187^Os/^188^Os) stratigraphy to reconstruct the ^187^Os/^188^Os composition of seawater over the event (see Supplemental Information).

The ^187^Os/^188^Os composition of seawater (^187^Os/^188^Os_sw_) reflects the sources of osmium to the ocean: rivers that drain continents (^187^Os/^188^Os_cont_ ≈ 1.4) and aeolian dust (^187^Os/^188^Os_aeol_ ≈ 1.04) represent a radiogenic end-member, and alteration of juvenile ocean crust or from the mantle (^187^Os/^188^Os_m_ ≈ 0.12) and cosmic dust/bolides (^187^Os/^188^Os_cos_ ≈ 0.12) represent an unradiogenic end-member^16^ (SI Fig. 1 and Supplemental Information). The flux of cosmic and aeolian dust represents a small fraction of the global input of osmium to the oceans and does not readily dissolve in seawater, and therefore does not appreciably affect the ocean’s ^187^Os/^188^Os_sw_ composition^16, 17^. The present-day ^187^Os/^188^Os_sw_ (~1.06) reflects the relatively greater input of continental-derived osmium to the ocean as compared to mantle-sourced osmium. Importantly, the short residence time of osmium in the oceans (~10^3^–10^4^ yr)^18^ permits the osmium isotope system to record ephemeral changes in global weathering patterns on the order of 10^3^ to 10^5^ years in the geological record^19^.

**Figure 2:**
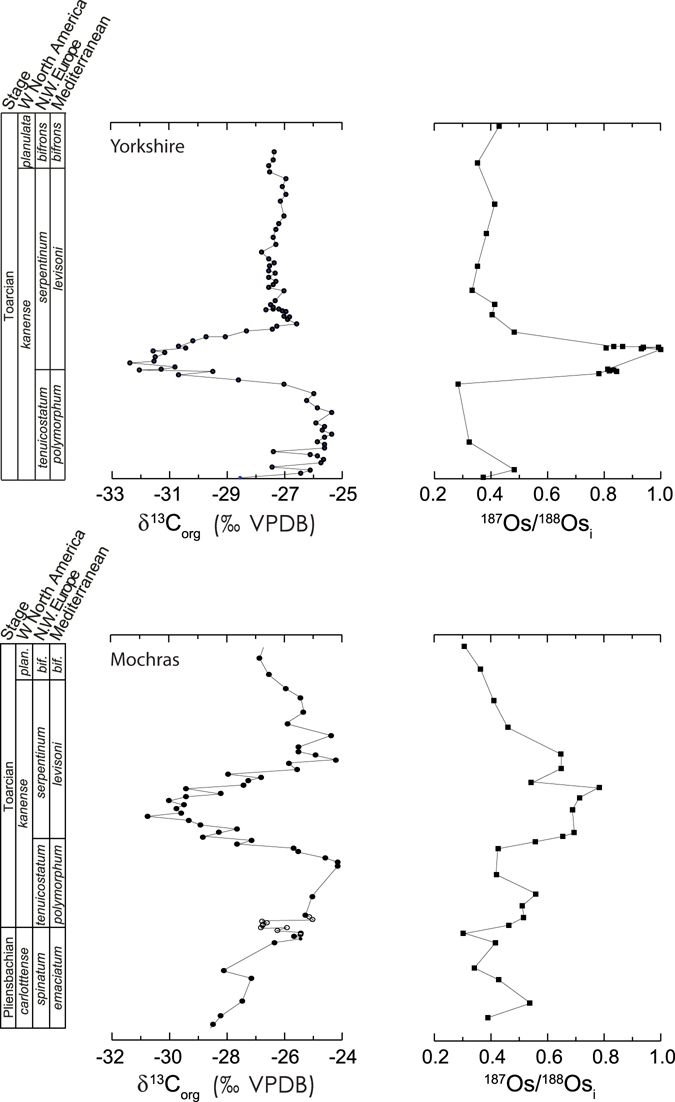
Records of the osmium isotope excursion across the T-OAE CIE from Yorkshire, United Kingdom^20^ and the Mochras borehole^23^. The Yorkshire dataset was originally interpreted to represent a 400–800% increase in continental weathering rates^20^; however, other interpretations suggests that the radiogenic values during the *exaratum* ammonite subzone were caused by hydrographic restriction^21, 22^. The close palaeogeographic proximity between these two sites, coupled with their significantly different ^187^Os/^188^Os_i_ values suggests a regional influence on ^187^Os/^188^Os_sw_ values in the European epicontinental seaway during the T-OAE.

The ^187^Os/^188^Os compositions of organic-rich sediments are known to record the ^187^Os/^188^Os composition of contemporaneous seawater^19^, and serve as an archive of the past marine osmium isotope compositions. A previous osmium isotope study of the T-OAE interval from a sedimentary succession in the Cleveland Basin of Yorkshire, United Kingdom indicates that, during the event, there was a concomitant, transient increase of ^187^Os/^188^Os_sw_ values by 0.7^20^ (Fig. 2). This record was originally interpreted to be the result of an increase in continental weathering rates of 400 to 800%^20^. However, it has been suggested that these data reflect regional climatic changes where enhanced local runoff influenced the ^187^Os/^188^Os_sw_ composition of the European epicontinental sea, which the Cleveland Basin was part of (Fig. 1), and therefore the ^187^Os/^188^Os record does not reflect a global weathering signal^21^. Key to this dispute is whether the Cleveland Basin was significantly hydrographically restricted so the local ^187^Os/^188^Os_sw_ signal could be modified^21, 22^. A recently published osmium isotope record across the T-OAE from the Mochras borehole^23^, located in nearby Wales, displays a much less pronounced excursion of 0.4 during the T-OAE interval (Fig. 2), which further suggests that geochemical changes recorded in the Cleveland Basin were likely influenced by regional climatic and oceanographic dynamics^18, 24, 25^.

**Figure 3:**
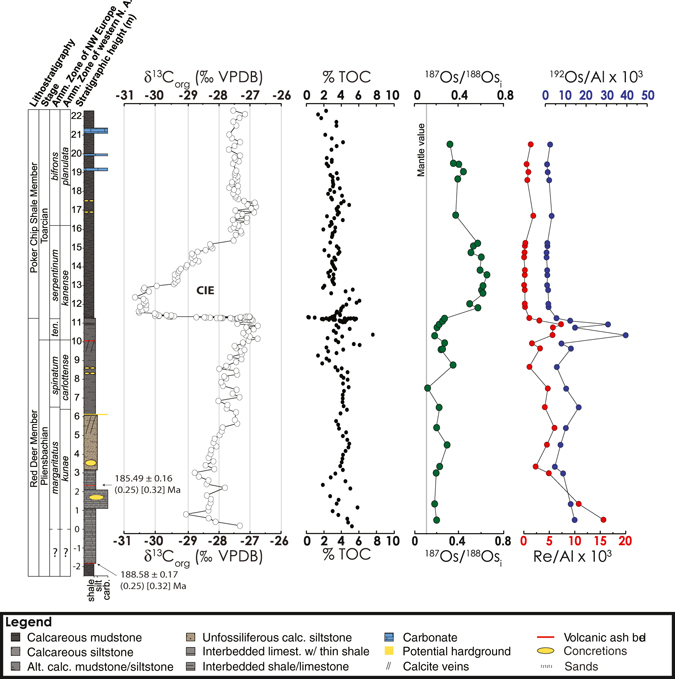
Chemostratigraphy of the Lower Jurassic Fernie Formation from East Tributary of Bighorn Creek, Alberta. δ^13^C_org_ = organic carbon isotopic compositions from ref. 15. ^187^Os/^188^Os_i_ = initial osmium isotopic composition of organic-rich sediments. Lithostratigraphic members of the Fernie Formation, Stages of the Jurassic, and ammonite zonations for both northwestern Europe and western North American shown to the left of the stratigraphic column (refer to ref. 15 for the details of their placements). Vertical gray line in ^187^Os/^188^Os_i_ record is the end-member ^187^Os/^188^Os_m_ value of ~0.12. We report new single zircon U-Pb CA-ID-TIMS ages of 188.58 ± 0.17 (0.25) [0.32] Ma in the bentonite at −1.9 meters and 185.49 ± 0.16 (0.25) [0.32] Ma in the bentonite at 2.35 meters, located in the *margaritatus* Zone of NW Europe or the *kunae* Zone of western NA (see Methods and SI Data 2).

To resolve whether the transient increases in ^187^Os/^188^Os observed across the T-OAE were indeed a global signal, we have investigated the osmium isotope record from the Lower Jurassic Fernie Formation of the Western Canada Sedimentary Basin located in present-day western Alberta (Fig. 1). This new location was situated on the eastern margin of the ocean of Panthalassa and therefore was located in a different ocean basin from the previously studied Yorkshire and Mochras sites (Figs 1 and 2). Ammonite biostratigraphy and carbon isotope stratigraphy of the Fernie Formation at East Tributary of Bighorn Creek has identified the upper Pliensbachian to middle Toarcian interval and the T-OAE CIEs^15, 26–28^. Zircon U-Pb dates from two bentonites located near the base of the section also provide temporal constraint and an age model for the section (Fig. 3; see Methods and Supplementary Data). Importantly, the entire interval of the East Tributary succession contains organic-rich strata (2–8% TOC; Figs 1 and 3)^15^, and thus represents an ideal location to reconstruct the global ^187^Os/^188^Os_sw_ over the T-OAE interval (see Supplemental Information).

## Results

### ^187^Os/^188^Os_i_ record from North America

The high-resolution initial ^187^Os/^188^Os (^187^Os/^188^Os_i_) record of the East Tributary succession (see Supplemental Information) displays extremely unradiogenic values (^187^Os/^188^Os_i_ ≈ 0.25) in the Pliensbachian and Lowest Toarcian, followed by a prominent radiogenic excursion (^187^Os/^188^Os_i_ ≈ 0.6) during the Toarcian CIEs (Fig. 3). The ^187^Os/^188^Os_i_ values decrease after the Toarcian CIE and asymptotically approach ~0.4 (Fig. 3; see Supplemental Information). Locally at East Tributary, aluminum and titanium concentrations increase 3-fold during the ^187^Os/^188^Os_i_ excursion and remain high for the rest of the record (see Fig. 3 and SI Dataset 1), which suggests a local increase in the contribution of continentally derived materials during the event. However, their concentrations remain high as ^187^Os/^188^Os_i_ values decrease after the Toarcian CIE, which suggests a minimal influence of a detrital component of rhenium and osmium to the osmium isotopic signature (see Fig. 3, Methods, and SI Dataset 1).

## Discussion

### Comparison of Early Jurassic ^187^Os/^188^Os_i_ records

Other marine ^187^Os/^188^Os_i_ records from the Lower Jurassic (Hettangian through Toarcian stages) generally show unradiogenic values^20, 23, 29, 30^. These are likely related to relatively elevated inputs of unradiogenic osmium from the weathering of the Central Atlantic Magmatic Province (CAMP) and the alteration of juvenile oceanic lithosphere or direct injection of mantle-derived osmium from initial opening of the North Atlantic^31^. The Upper Pliensbachian portion of our record from northeastern Panthalassa has broadly similar values to those observed in the European epicontinental sea^20, 23, 29^, which suggests they are representative of the global ^187^Os/^188^Os_sw_ values, and indicative of a well-mixed Early Jurassic ocean. Further, the East Tributary ^187^Os/^188^Os_i_ record shows a similar pattern to the other available records during the interval that contains the T-OAE^20, 23^. All the sites record an excursion to higher ^187^Os/^188^Os_i_ values that follow the falling limb of the Toarcian negative CIE. This trend is followed by a return to lower ^187^Os/^188^Os_i_ values after the rising limb of the negative CIE. However, in all cases ^187^Os/^188^Os_i_ declines to values slightly higher than those observed before the excursion.

While all the ^187^Os/^188^Os_i_ records display a similar overall pattern, their ^187^Os/^188^Os_i_ values differ. The Yorkshire and East Tributary datasets have similar ^187^Os/^188^Os_i_ values before and after the T-OAE (~0.3 and ~0.4, respectively); however, the Yorkshire dataset shows an excursion to significantly more radiogenic values (^187^Os/^188^Os_i_ ≈ 1) during the T-OAE^20^ (Fig. 2). The Mochras data show higher ^187^Os/^188^Os_i_ values just before the T-OAE CIE (~0.4), which increase to an acme of 0.8 during the T-OAE, and decrease to ~0.3 after the event^23^ (Fig. 2). While the absolute ^187^Os/^188^Os_i_ values differ between the sites, the magnitude of the excursions at East Tributary and Mochras are similar at 0.4, and are almost half the magnitude observed at Yorkshire (0.7).

The differences observed between the ^187^Os/^188^Os_i_ records at East Tributary, Mochras, and Yorkshire suggest there were regional differences in ^187^Os/^188^Os_sw_ during the studied interval. These differences likely represent local processes such as differing degrees of hydrographic restriction from the open ocean and the amounts of local runoff and its ^187^Os/^188^Os composition. However, the similarity in the magnitude of the excursions recorded at East Tributary and Mochras suggest this likely represents the global record of change during the T-OAE. This observation, coupled with the more extreme ^187^Os/^188^Os_i_ excursion record at Yorkshire, supports the suggestion that the Yorkshire ^187^Os/^188^Os_sw_ record was influenced by a local riverine input of radiogenic osmium during the T-OAE^21^, and the East Tributary and Mochras records are more representative of global osmium seawater chemistry

With these observations in mind, we advocate, when possible, analyzing osmium isotope records from coeval stratigraphic successions deposited in different sedimentary and ocean basins^18, 24–26^ before attempting to interpret them as a global signal. This methodology is especially important regarding palaeoceanographic studies on intervals older than the Cretaceous since the preserved records are predominantly from continental margin and epicontinental successions, where geochemical signatures have a greater potential to be modified by local processes.

### Quantifying the Early Jurassic marine osmium cycle

**Figure 4:**
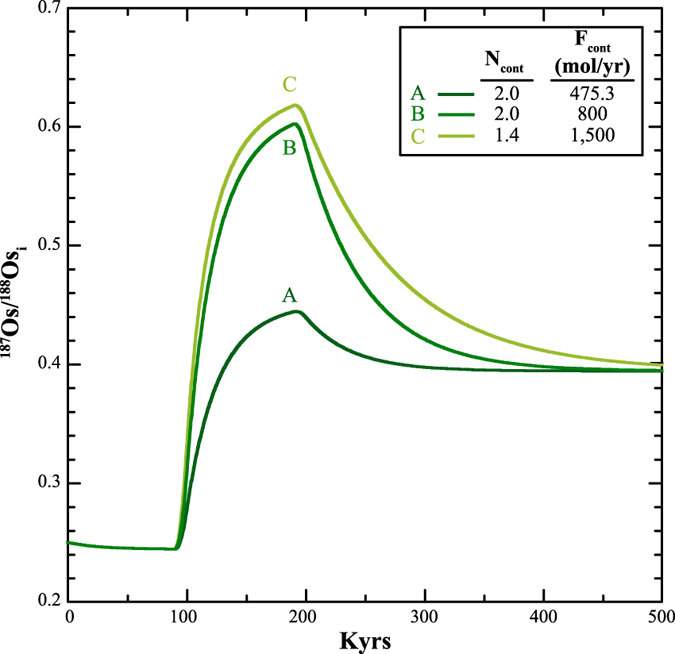
Examples of the modelled osmium isotopic composition of the ocean over the T-OAE. (**A**) For this model run, the osmium isotopic composition of the continental input was increased to 2.0 and the flux of osmium from continents was increased two-fold (475.3 mol/yr) during the Toarcian OAE. This resulted in the seawater osmium isotope values to increase to 0.44, which does not reproduce the observed osmium isotope excursion observed at East Tributary. (**B**) Model run where the osmium isotopic composition and flux of the continental input of osmium was increased to 2.0 by ~3.4x respectively. This model run reproduced the osmium isotope excursion. (**C**) The osmium isotope composition of the continental input of osmium was kept at 1.4 during the Toarcian OAE, but the flux of osmium from continents was increased by ~6.3x to reproduce the osmium isotope excursion.

To gain a more quantitative measure of the changes in the marine osmium cycle during the Toarcian we employed a numerical box model that simulates the osmium inventory of the ocean and its isotopic composition (see Supplemental Information). Specifically, we test whether the osmium isotope excursion associated with the T-OAE (~300–500 kyr in duration)^31, 32^ can be reproduced by a transient increase in the weathering input of radiogenic osmium to the ocean. We also explored other situations that may have potentially driven the observed T-OAE osmium isotope record, but are likely implausible, such as decreasing the input flux of mantle-derived osmium to zero (see Table 1 for values explored and Supplemental Information for a discussion of these cases). Overall, the numerical model results show that the osmium isotope excursion can be reproduced by a transient three- to six-fold increase in the input of continental-derived osmium to the oceans over 100 to 200 kyr^31, 32^ (Fig. 4; more details of the modelling results including sensitivity tests can be found in the Supplemental Information).

**Table 1:** Range of parameters explored modelling the osmium isotope excursion associated with the Toarcian Oceanic Anoxic Event in the East Tributary and Yorkshire sections.

Model parameter	Pre- and post-T-OAE steady state	OAE state
M_SW_^a^	10^5^ to 10^9^	10^5^ to 10^9^
F_cont_^b^	238 to 524	238 to 5,500
N_cont_	1.4 to 2.0	1.4 to 5.0
F_m_^b^	1,925 to 2,212	0 to 2,212
N_m_	0.12	0.12
Duration^c^		300 to 500

^a^Reservoir unit is mol Os.

^b^Flux units are mol/yr Os.

^c^Duration unit is kiloyear (kyr).

Changes in the ^187^Os/^188^Os_cont_ to more radiogenic values through the differential weathering of lithologies such as shales and cratonic rocks^33–35^ could have played a role in the T-OAE osmium isotope record. We investigated the potential effect this change would have on the osmium budget during the event by running simulations where we elevated ^187^Os/^188^Os_cont_ from 1.4 to 2 (see Supplemental Information for a discussion of the choice of the maximum ^187^Os/^188^Os_cont_ value). In these simulations, a nearly three-fold increase of the input of continental-derived osmium to the oceans was still necessary to reproduce the excursion (Fig. 4), regardless of timescale used, and solely increasing ^187^Os/^188^Os_cont_ to reasonable values cannot reproduce the observed excursion (see Supplemental Information). Given the plausible proposition of the changing composition of the continental weathering flux, we conservatively suggest that T-OAE weathering rates increased by as much as three-fold.

A potential source of radiogenic, continentally derived osmium was the remnants of the Central Pangaean Mountains, a Himalayan-scale mountain belt in eastern North America and northwestern Africa. This mountain belt was positioned at tropical and subtropical latitudes in the Early Jurassic (Fig. 1). The rifting of Pangaea during the Late Triassic and Early Jurassic would have exposed the core of the mountain range leaving this material open to weathering or erosion. General circulation models predict large increases in the air temperature and runoff during the T-OAE in the geographic region that contained these mountains^36^. These regional climatic changes would have facilitated enhanced chemical weathering, and makes this mountain belt a plausible source of the enhanced input of osmium to the oceans advocated here.

The weathering of organic-rich rocks and sediments would be another plausible way to raise the isotopic composition of the continental weathering flux, but also results in a net release of CO_2_ to the atmosphere^37^. However, enhanced continental runoff would also have increased nutrient delivery and stimulated primary productivity in aquatic environments leading to increased hypoxia, anoxia, and potentially euxinia^5^. Elevated burial of organic matter in these environments would have sequestered much more atmospheric CO_2_ than that associated with any black shale weathering, which we suggest represent only a fraction of the continental materials that were predominantly weathered during the event.

### Differences in the osmium isotope response between OAE events

A striking feature of the ^187^Os/^188^Os records during the Mesozoic OAEs is the directionality of their excursions. The T-OAE records show a positive ^187^Os/^188^Os excursion, whereas the onset of the Cretaceous OAE 1a and OAE 2 both display negative excursions. The difference in the ^187^Os/^188^Os response to these events most likely lies in the environment where the LIPs were emplaced. The Cretaceous events are associated with subaqueous emplacements of the Ontong Java Plateau (OAE 1a) and the Caribbean and High Arctic LIPs (OAE 2). Emplacement of these LIPs would have supplied large amounts of unradiogenic, mantle-derived osmium directly into the oceans from weathering of basalts on the seafloor, resulting in osmium isotope excursions to nonradiogenic values^25, 38–40^.

The T-OAE, on the other hand, is associated with a subaerial emplacement of the Karoo-Ferrar LIP at high latitudes (Fig. 1), where the semi-arid climate would have made the relative weathering potential of this material low. In contrast to the younger OAEs, the Toarcian ^187^Os/^188^Os_i_ records reflect enhancement of the weathering of continental materials facilitated by the injection of greenhouse gases into the atmosphere and subsequent climate changes. Notably, delivery of osmium from the Karoo-Ferrar LIP would have also been delayed, as compared to the Cretaceous LIPs. However, if weathering of the Karoo-Ferrar LIP was a significant source of osmium to the oceans during the T-OAE, then its lower ^187^Os/^188^Os compositions^41–45^ would necessitate an even greater contribution of continental material to generate the observed ^187^Os/^188^Os_i_ excursion.

### Implications and Conclusions

Based on the osmium isotope records and our modelling results, the transient increase in continental weathering rates during the T-OAE may be one of the largest observed during the Phanerozoic. Chemical weathering rates are also suggested to have significantly increased across the Permian-Triassic boundary^46^, Triassic-Jurassic boundary^47, 48^, and the Paleocene-Eocene Thermal Maximum^49^, all of which are associated with intervals of global warming, environmental deterioration, and extinction events^50^. The rapid response of the osmium isotope system during the T-OAE, as well as during other OAEs^38–40^, indicates that chemical weathering feedbacks may respond to episodes of rapid climatic warming on short timescales (10^3^–10^6^ years) and lead to a net drawdown of atmospheric CO_2_^5^. Enhanced continental runoff would also have increased nutrient delivery and stimulated primary productivity in nearshore environments, leading to increased marine hypoxia, anoxia, and potentially euxinia^5^. CO_2_ would also have been sequestered through the deposition of organic-rich sediments in marine and lacustrine settings^5, 6, 51^.

In the case of the Toarcian OAE, increased weathering likely played a critical role in reversing the enhanced greenhouse state induced by Karoo-Ferrar magmatism. As atmospheric CO_2_ was consumed through these mechanisms, global temperatures would have declined^5, 20^. As modern atmospheric CO_2_ levels continue to increase at rates much higher than any point during the Cenozoic^52^, increased weathering, through the chemical and physical weathering feedbacks and stimulation of primary production and subsequent organic matter burial, may eventually act as a negative feedback to global warming, although on timescales much longer than what is necessary to mitigate the immediate environmental and ecological deterioration due to this warming^53^.

## Methods

### δ^13^C and total organic carbon analysis

δ^13^C and total organic carbon (TOC) were measured from each sample for rhenium, osmium, and trace metals (see below). The samples were prepared and analysed using the same methods from ref 15.

### Rhenium and osmium isotopic analysis

In order to isolate primarily the hydrogenous rhenium and osmium from our samples, and minimize the removal of detrital rhenium and osmium, we followed the procedures of ref. 54. Between ~0.25 and 1 g of sample powder (dependent upon previously measured rhenium abundances via inductively-coupled plasma mass spectrometry) were digested with a known amount of ^185^Re and ^190^Os tracer (spike) solutions in 8 mL of a CrO_3_-H_2_SO_4_ solution; this reaction occurred in sealed Carius tubes, which were heated incrementally to 220 °C for 48 hours. The tubes were allowed to cool before opening. The osmium was immediately isolated and purified from the acid medium by solvent extraction using chloroform. This step was followed by the back reduction of Os from the chloroform into HBr. The Os fraction was further purified by micro-distillation. Rhenium was purified from the remaining CrO_3_-H_2_SO_4_ solution by a NaOH-Acetone solvent extraction^55^ and further purified using anion exchange chromatography. The purified Re and Os fractions were then loaded onto Ni and Pt filaments, respectively, and analysed for their isotopic composition using negative thermal-ionization mass spectrometry (NTIMS)^56, 57^ using a Thermo Scientific TRITON mass spectrometer with static Faraday collection for Re and ion-counting using a secondary electron multiplier in peak-hopping mode for Os. In-house Re and Os solutions were continuously analysed during the course of this study to ensure and monitor long-term mass spectrometry reproducibility. A 125 pg aliquot of the Re std solution and a 50 pg aliquot of DROsS yield ^185^Re/^187^Re values of 0.5983 ± 0.002 (1 SD, n = 6) and ^187^Os/^188^Os values of 0.16089 ± 0.0005 (1 SD, n = 8), respectively; both are identical to previously reported values^57^. The measured difference in ^185^Re/^187^Re values for the Re std solution and the accepted ^185^Re/^187^Re value (0.5974)^58^ is used for mass fractionation correction of the Re sample data. All Re and Os data are oxide and blank corrected. Procedural blanks for Re and Os in this study were 12 ± 3 pg/g and 0.07 ± 0.05 pg/g, respectively, with an ^187^Os/^188^Os value of 0.25 ± 0.15 (n = 4). The ^187^Re/^188^Os and ^187^Os/^188^Os uncertainties are determined through full propagation of uncertainties, including those in weighing, mass spectrometer measurements, spike calibrations, blank abundances and reproducibility of standard values.

### Trace metal analysis

In order to compare the changes in [Re] and [Os] to sedimentation patterns across the T-OAE, we also analysed the concentrations of aluminum and titanium in each sample, which are used to estimate the contribution of terrigenous input to a sedimentary basin^59, 60^ (see Fig. 3 and SI dataset). Approximately 0.05 g of powder was added to a teflon beaker, followed by the addition of 4 mL of a 50:50 mixture of concentrated HCl and concentrated HNO_3_. This solution was placed inside a (CEM MARS 5) microwave assisted digestion system and run until all organic material had broken down at a temperature of 150 °C. The samples were then dried down and the silicates were dissolved using 4:1 HNO_3_ to HF, dried down, and re-dissolved in 5% HNO_3_ solution. A 100 μL solution split was spiked with an internal standard to measure elemental abundances using an Agilent 7500cs inductively-coupled plasma mass spectrometer in He and H mode. Internal standard was used to correct the samples for machine drift. International standards USGS SCO-1 and SDO-1 were also measured and had a reproducibility of ± 5%.

### U-Pb analysis of zircons

CA-TIMS procedures described here are modified from refs 61–63. After rock samples have undergone standard mineral separation procedures zircons are handpicked in alcohol. The clearest, crack- and inclusion-free grains are selected, photographed, and then annealed in quartz glass crucibles at 900 °C for 60 hours. Annealed grains are transferred into 3.5 mL PFA screwtop beakers, ultrapure HF (up to 50% strength, 500 μL) and HNO_3_ (up to 14 N, 50 μL) are added and caps are closed finger tight. The beakers are placed in 125 mL PTFE liners (up to four per liner) and about 2 mL HF and 0.2 mL HNO_3_ of the same strength as acid within beakers containing samples are added to the liners. The liners are then slid into stainless steel Parr™ high pressure dissolution devices, which are sealed and brought up to a maximum of 200 °C for 8–16 hours (typically 175 °C for 12 hours). Beakers are removed from liners and zircon is separated from leachate. Zircons are rinsed with > 18 MΩ.cm water and subboiled acetone. Then 2 mL of subboiled 6 N HCl is added and beakers are set on a hotplate at 80°–130 °C for 30 minutes and again rinsed with water and acetone. Masses are estimated from the dimensions (volumes) of grains. Single grains are transferred into clean 300 μL PFA microcapsules (crucibles), and 50 μL 50% HF and 5 μL 14 N HNO_3_ are added. Each is spiked with a ^233–235^U-^205^Pb tracer solution (EARTHTIME ET535), capped, and again placed in a Parr liner (8–15 microcapsules per liner). HF and nitric acids in a 10:1 ratio, respectively, are added to the liner, which is then placed in a Parr high pressure device and dissolution is achieved at 220 °C for 40 hours. The resulting solutions are dried on a hotplate at 130 °C, 50 μL 6 N HCl is added to microcapsules and fluorides are dissolved in high-pressure Parr devices for 12 hours at 180 °C. HCl solutions are transferred into clean 7 mL PFA beakers and dried with 2 μL of 0.5 N H_3_PO_4_. Samples are loaded onto degassed, zone-refined Re filaments in 2 μL of silicic acid emitter^64^.

Isotopic ratios are measured with a modified single collector 354S (with Sector 54 electronics) thermal ionization mass spectrometer equipped with analogue Daly photomultipliers. Analytical blanks are 0.2 pg for U and up to 1.9 pg for Pb. U fractionation was determined directly on individual runs using the EARTHTIME ET535 mixed ^233–235^U-^205^Pb isotopic tracer and Pb isotopic ratios were corrected for fractionation of 0.25 ± 0.03%/amu, based on replicate analyses of NBS-982 reference material and the values recommended by ref. 65. Data reduction employed the excel-based program of ref. 66. Standard concordia diagrams were constructed and regression intercepts, weighted averages calculated with Isoplot^67^. Unless otherwise noted all errors are quoted at the 2-sigma or 95% level of confidence. Isotopic dates are calculated with the decay constants λ_238_ = 1.55125E-10 and λ_235_ = 9.8485E-10 (ref. 68) and a ^238^U/^235^U ratio of 137.88. EARTHTIME U-Pb synthetic solutions are analysed on an on-going basis to monitor the accuracy of results.

Five single zircon grains from the bentonite at −1.9 meters in the East Tributary section (see Fig. 3) were analysed by the uranium-lead chemical abrasion isotope dilution thermal ionization mass spectrometry technique (U-Pb CA-ID-TIMS). A weighted mean ^206^Pb/^238^U age of 188.58 ± 0.17 (0.25) [0.32] Ma, (MSWD = 0.89) is based on concordant and overlapping results for three of the analysed grains (see SI Dataset 2). Older results for the other two grains suggest that they are xenocrysts and/or contain inherited cores. It is important to note that this bentonite has a previously published multigrain U-Pb ID-TIMS age of 188.3 + 1.5/−1 Ma^69^.

Five single zircon grains from the bentonite at 2.35 meters in the East Tributary section (see Fig. 3) were analysed by the U-Pb CA-ID-TIMS technique. A weighted mean ^206^Pb/^238^U age of 185.49 ± 0.16 (0.25) [0.32] Ma, (MSWD = 1.17) is based on concordant and overlapping results for three of the analysed grains (see SI Dataset 2). Older results for the other two grains, one of which is discordant, suggest that they are xenocrysts and/or contain inherited cores.

### Age model and calculation of ^187^Os/^188^Os_i_

The age model (see below) is constructed using a single grain U-Pb CA-ID-TIMS age of 188.58 ± 0.17 (0.25) [0.32] Ma from approximately two meters below the lowest interval with carbon isotope data in the East Tributary section^15^ and a single grain U-Pb CA-ID-TIMS age of 185.49 ± 0.16 (0.25) [0.32] Ma (see above) located at 2.35 meters in the section (see Fig. 3). Linear interpolation was used to calculate ages between the bentonites layers and between the age assigned for the Toarcian CIE. The onset of the CIE is placed at 183.1 Ma, with a total duration of 300 kyr^31^. Sedimentation rates are also assumed to remain constant after the Toarcian CIE. The initial osmium isotopic composition of the oceans (^187^Os/^188^Os_i_) was calculated using the following equation and the ^187^Re decay constant from ref. 70:

1 $$\frac{{}^{187}Os}{{}^{188}O{s}_{i}}=\frac{{}^{187}Os}{{}^{188}Os}-(\frac{{}^{187}{R}{e}}{{}^{188}Os}\times {e}^{(1.666\times {10}^{-11}.{a}^{-1}\times age\times 1000000)}-1)$$

This equation accounts for the ^187^Os produced after deposition by the decay of ^187^Re. As stated above, the age component was derived from U-Pb ages from this succession (this study) and previously published dates for the age and estimated duration of the Toarcian CIE^31^. Furthermore, if a longer 500-kyr duration^32^ is assigned to the T-OAE CIE, the calculated ^187^Os/^188^Os_i_ values do not change significantly and our interpretations do not change (see Supplemental Information).

## Electronic supplementary material

Supplementary Information

Dataset 1

Dataset 2
