# Supplementary material for: Evidence for rapid weathering response to climatic warming during the Toarcian Oceanic Anoxic Event
Source: Sci Rep. 2017 Jul 10;7:5003. doi: 10.1038/s41598-017-05307-y (PMC5504049; doi:10.1038/s41598-017-05307-y)
Supplement: Supplementary file 1 — Supplementary Information [file 41598_2017_5307_MOESM1_ESM.doc]

Supplemental Information for:

Evidence for rapid weathering response to climatic warming during the Toarcian Oceanic Anoxic Event

**Theodore R. Them II^1,2*^, Benjamin C. Gill^1^, David Selby^3^, Darren R. Gröcke^3^, Richard M. Friedman^4^, and Jeremy D. Owens^2^**

**SUPPLEMENTAL INFORMATION**

**Estimating continental and oceanic contributions of osmium to the global oceans**

In order to better determine what processes could result in the observed osmium isotope excursion, we built a forward box model of the osmium cycle (SI Fig. 1). The change in the osmium isotope composition of the ocean was calculated using the following equation:

$\frac{dN_{SW}}{dt}=\frac{F_{cont}\left(N_{cont}-N_{SW}\right)+F_{M}\left(N_{M}-N_{SW}\right)}{M_{SW}}$ (1)

where dN_SW_/dt represents the change in the osmium isotopic composition of seawater with time, N_SW_ represents the osmium isotopic composition of seawater; F_cont_ represents the flux of radiogenic, continental osmium from rivers, and N_cont_ represents its isotopic composition; F_m_ represents the flux of unradiogenic, mantle osmium from the alteration of juvenile oceanic crust and hydrothermal fluids, and N_m_ represents the osmium isotopic composition of this input; and M_SW_ represents the global inventory of oceanic osmium.

We calibrated our model to the modern osmium cycle and utilized the flux estimate of osmium to the oceans from the riverine input of 1800 moles Os yr^-1^ with an isotopic composition of 1.4 and an ocean inventory of 7x10^7^ moles Os (Peucker-Ehrenbrink & Ravizza, 2000 and references therein). In order for the ocean reservoir to maintain isotopic steady state (dN_SW_/dt = 0) at the modern marine isotope composition of 1.06, a flux of osmium from the mantle of 650 moles Os yr^-1^ with an isotope composition of 0.12 is required. To maintain mass balance, the flux of osmium sequestered in sediments was set to 2450 moles Os yr^-1^.

**Estimating continental and oceanic contributions of osmium to the Jurassic global oceans**

In the following calculations, we initially set the total Jurassic input of osmium to the oceans at the modern estimate of 2450 moles Os yr^-1^ (previously calculated using values from Peucker-Ehrenbrink and Ravizza, 2000 and the steady state model above). In order to reach a pre-Toarcian OAE steady-state ^187^Os/^188^Os_sw_ value of ~0.25, the continental and mantle inputs of osmium to the ocean were set at 238 moles Os yr^-1^ (^187^Os/^188^Os_cont_ = 1.4) and 2,210 moles Os yr^-1^ (^187^Os/^188^Os_m_ = 0.12), respectively. If the global input of osmium to the Jurassic oceans was much higher or lower than today, then these fluxes can be scaled accordingly in order to maintain isotopic steady state. We also note, the different pre-event ^187^Os/^188^Os_sw_ observed at Mochras could be achieved with slightly higher F_cont_ and/or ^187^Os/^188^Os_cont_. Using the above isotopic compositions, the unradiogenic, F_m_ was roughly 90% of the flux of osmium to the oceans and the radiogenic, F_cont_ was only approximately 10% before the Toarcian OAE. As stated above, we have not included the flux of osmium from cosmic dust or aeolian dust since these are thought to be minor inputs to the ocean, and the cosmic flux is generally assumed constant (Peucker-Ehrenbrink, 1996).

Based on our knowledge of the marine osmium cycle, several scenarios could have potentially produced the Toarcian osmium isotope record recorded at the East Tributary section based on the directionality of the excursion. These include transiently 1) increasing F_cont_, 2) increasing N_cont_, 3) increasing both F_cont_ and N_cont_, 4) decreasing F_m_. We therefore conducted a series of simulations and sensitivity tests using the model in order to identify scenarios that produced acceptable results (i.e. reproduced the magnitude and duration of the observed osmium isotope excursion). Across our simulations, durations of 100 to 300 kyrs for the transient change in the osmium cycle produced positive excursions with durations of 300 to 500 kyrs (SI Fig. 2) and are consistent with estimates for the duration of the overall osmium isotope excursion (Sell et al., 2014; Boulila et al., 2014).

Solely increasing F_cont_ or increasing both F_cont_ and N_cont_ produced solutions that reproduced the observed Os isotope record. For an example of scenario 1, increasing the F_cont_ for 100 kyr from 238 mol Os yr^-1^ to 1,500 mol Os yr^-1^ reproduced the magnitude and timing of the observed isotope excursion. Increasing N_cont_ in conjunction with F_cont_ decreases the needed increase in F_cont_ (see discussion below on solely changing the isotopic composition of the riverine flux). For an example of scenario 3, changing N_cont_ from 1.4 to 2.0, and increasing the F_cont_ from 238 mol Os yr^-1^ to 800 mol Os yr^-1^ for 100 kyr, resulted in an acceptable solution. Broadly across our simulations, increases in F_cont_ of 238 to 1,500 moles per year depending model conditions (e.g., the value(s) of N_cont_) could reproduce the Toarcian osmium isotope excursion.

Other scenarios also produced acceptable numerical solutions; however, these represent geologically unlikely scenarios. For example, it is possible to reproduce the isotope excursion by changing only the isotopic composition of osmium entering the oceans from continents. However, this requires that, at a minimum, ^187^Os/^188^Os_cont_ values transiently increase to 5. The highest recorded modern riverine ^187^Os/^188^Os values were found within Mackenzie River basin at 3 – 4.5, and these compositions were isolated to only a few tributaries within the watershed. These tributaries do, however, cause the Mackenzie River to be slightly more radiogenic (^187^Os/^188^Os = 1.5-1.7) than the world river average ^187^Os/^188^Os of 1.4 (Huh et al., 2004). Therefore, we conclude that it is unlikely that global ^187^Os/^188^Os_cont_ values increased to values much greater than 2 during the T-OAE (Cohen et al., 2004).

Simulations where F_m_ was transiently reduced did not produce acceptable solutions. For example, eliminating the mantle flux for 100 kyrs yields an excursion with a maximum value of only 0.55. Further, this is also an unrealistic scenario as there is no reasonable way to explain why the weathering of unradiogenic mafic materials (CAMP basalts, juvenile oceanic crust, etc.) would cease during the event.

It is also important to note that decreasing the M_SW_ inventory does not significantly alter the needed increase in F_cont_ or N_cont_ necessary to generate the osmium isotope excursion (SI Fig. 3 displays example sensitivity tests of varying M_SW_). This is due to the relatively short residence time of Os (Toarcian residence times explored here: 10 to 90 kyrs) in the ocean as compared to the duration of the osmium isotope excursion. This is important because with the expansion of marine anoxia during the event, it is plausible that the Os reservoir was significantly reduced. Reducing M_SW_ does, however, affect how quickly N_SW_ reaches its peak value (SI Fig. 3). However, simulations with M_SW_ less than a third of the modern marine inventory produced osmium isotope excursions with rising limbs that were shorter than the minimum estimated durations (~100 kyrs) inferred from the Toarcian osmium isotope records (Cohen et al., 2004; Percival et al., 2016; this study). This, therefore, places a limit on the potential decrease in M_SW_ due to the expansion of anoxia during the T-OAE. Increasing M_SW_ over an order of magnitude greater than modern reservoir produced rising limbs that were too long (greater than 250 kyrs) or the excursion did not reach the observed peak in ^187^Os/^188^Os. These sensitivity tests suggest that M_SW_ was within an order of magnitude of the size of modern marine reservoir.

We also simulated the effects of changing the duration of the changes in the osmium cycle would have on the duration of the osmium isotope excursion (SI Fig. 2). We tested three scenarios, a) transiently and instantaneously increasing F_cont_ and N_cont_ for 100 kyrs followed by a return to a new steady state, b) increasing F_cont_ and N_cont_ in 20 kyrs steps over 100 kyr, letting F_cont_ and N_cont_ remain constant for 100 kyr, and then decreasing F_cont_ and N_cont_ in 20 kyr steps over 100 kyrs (300 kyrs of total perturbation), and c) increasing F_cont_ and N_cont_ in 40 kyr steps over 200 kyr, letting F_cont_ and N_cont_ remain constant for 100 kyr, and then decreasing F_cont_ and N_cont_ in 40 kyr steps over 200 kyr (500 kyrs of total perturbation). The 100-kyr and 300-kyr perturbations produced excursions that satisfy the U-Pb estimation from South America (Sell et al., 2014), and the 500-kyr perturbation satisfies the astronomical calibration from Europe (Boulila et al., 2014).

We also reproduced the osmium isotope excursion from Yorkshire to test whether plausible scenarios could produce that osmium isotope record (Fig. 2 of main text) (Cohen et al., 2004). For example, increasing the flux of osmium from continents from 238 to 5,500 mol Os yr^-1^ for 100 kyr (using pre-OAE steady-state conditions calculated from the Alberta osmium dataset) can reproduce the magnitude and timing of the observed Yorkshire osmium isotope excursion (see SI Fig. 4). This constitutes an increase of ~2,200% above the pre-T-OAE riverine flux values. Also, changing the osmium isotopic composition of the continental end-member from 1.4 to 2.0, and increased the flux of continental-derived osmium from 238 to 2,100 mol Os yr^-1^ (an increase in riverine osmium delivery of ~800%) for 100 kyr resulted in an acceptable solution (see SI Fig. 4). However, both of these values require an extremely large (and likely unreasonable) increase in the riverine flux to the ocean if the Yorkshire dataset is indicative of a global signal. Therefore, it is unlikely that this record reflects the ^187^Os/^188^Os evolution of the global ocean and was probably modified by local/regional riverine inputs during the T-OAE (McArthur et al. 2008). As such, the long-term Yorkshire ^187^Os/^188^Os record is identical to that of the Mochras borehole (Percival et al., 2016) and northeastern Panthalassa (this study).

**SI FIGURE CAPTIONS**

SI Figure 1. The exogenic osmium cycle (modified from Peucker-Ehrenbrink and Ravizza, 2000). The major inputs of osmium to oceans are from the weathering of materials from the continents (^187^Os/^188^Os_cont_ ≈ 1.4) and the alteration of juvenile oceanic crust (^187^Os/^188^Os_m_ ≈ 0.12). Sequestration of the seawater inventory of osmium occurs during precipitation of iron-manganese crusts on the ocean bottom and through biological uptake associated with primary productivity and burial in sediments.

SI Figure 2. Examples of the modelled osmium isotopic composition of the ocean over the T-OAE when changing the duration of the T-OAE. **A)** For this model run, F_cont_ and N_cont_ were transiently and instantaneously increased for 100 kyr followed by a return to a new steady-state **B)** Model run where F_cont_ and N_cont_ were increased in 20-kyr steps over 100 kyr, F_cont_ and N_cont_ remained constant for 100 kyr, and then F_cont_ and N_cont_ were decreased in 20-kyr steps over 100 kyr, and **C)** Model run where F_cont_ and N_cont_ were increased in 40 kyr steps over 200 kyr, F_cont_ and N_cont_ remained constant for 100 kyr, and then F_cont_ and N_cont_ were decreased in 40 kyr-steps over 200 kyr. Model A required an increase in weathering rates of 230%, whereas model runs B and C required an increase in weathering rates of 215%. Therefore, changing the duration of the T-OAE does not significantly change our interpretations of increased weathering rates; it does, however, result in different overall amounts of osmium added into the ocean during the event.

SI Figure 3. Examples of the modeled osmium isotopic composition of the ocean over the T-OAE when only changing M_ocean_. **A)** For this model run, M_ocean_ was set to 7x10^5^ moles **B)** Model run where M_ocean_ was set to 7x10^6^ moles **C)** Model run where M_ocean_ was set to 7x10^7^ moles (modern M_ocean_ value) **D)** Model run where M_ocean_ was set to 7x10^8^ moles **E)** Model run where M_ocean_ was set to 7x10^9^ moles. F_cont_ and N_cont_ remained constant for each simulation, and a step function was used to increase and decrease both parameters for 100 kyr.

SI Figure 4. Examples of the modeled osmium isotopic composition of the ocean over the T-OAE in order to replicate the Yorkshire ^187^Os/^188^Os_i_ record. **A)** For this model run, the N_cont_ was constant (^187^Os/^188^Os_cont_ = 1.4) and the F_cont_ was increased to 5,500 mol/yr during the T-OAE. This resulted in the seawater osmium isotope values to increase to 1. **B)** Model run where N_cont_ was increased to 2.0 during the T-OAE, and F_cont_ was increased to 2,100 mol/yr. Both of these scenarios suggest an unrealistic increase in the amount of osmium delivered from the continents during the T-OAE.

**REFERENCE CITED FOR SUPPLEMENTARY INFORMATION**

Boulila, S. *et al.* Astronomical calibration of the Toarcian State: Implications for sequence stratigraphy and duration of the early Toarcian OAE. *Earth Plan. Sci. Lett.* **386**, 98–111 (2014).

Cohen, A.S. The rhenium-osmium isotope system: applications to palaeoenvironmental problems. *J. Geol. Soc. Lon.* **161**, 729–734 (2004).

Cohen, A.S., Coe, A.L., Harding, S.M. & Schwark, L. Osmium isotope evidence for the regulation of atmospheric CO_2_ by continental weathering. *Geology* **32**, 157– 160 (2004).

Esser, B.K. & Turekian, K.K. The osmium isotopic composition of the continental crust. *Geochim. Cosmochim. Acta* **57**, 3093–3104 (1993).

Huh, Y., Birck, J.-L. & Allègre, C.J. Osmium isotope geochemistry in the Mackenzie River basin. *Earth Plan. Sci. Lett.* **222**, 115–29 (2004).

McArthur, J.M., Algeo, T.J., van de Schootbrugge, B., Li, Q. & Howarth, R.J. Basinal restriction, black shales, Re-Os dating, and the Early Toarcian (Jurassic) oceanic anoxic event. *Paleoceanography* **23**, PA4217 (2008).

Percival, L.M.E., Cohen, A.S., Davies, M.K., Dickson, A.J., Hesselbo, S.P., Jenkyns, H.C., Leng, M.J., Mather, T.A., Storm, M.S. & Xu. W. Osmium isotope evidence for two pulses of increased continental weathering linked to Early Jurassic volcanism and climate change. *Geology* **44**, 759– 762 (2016).

Peucker-Ehrenbrink, B. Accretion of extraterrestrial matter during the last 80 million years and its effect on the marine osmium isotope record. *Geochim. Cosmochim.* *Acta* **60**, 3187–3196 (1996).

Peucker-Ehrenbrink, B. Comment on “Residence time of osmium in the oceans” by Rachel Oxburgh. *G^3^* **3**, 1–4 (2002).

Peucker-Ehrenbrink, B. & Ravizza, G. The marine osmium isotope record. *Terra Nova* **12**, 205–219 (2000).

Sell, B., Ovtcharova, M., Guex, J., Bartolini, A., Jourdan, F., Spangenberg, J.E., Vicente, J.-C. & Schaltegger, U. Evaluating the temporal link between the Karoo LIP and climatic—biologic events of the Toarcian Stage with high-precision U-Pb geochronology. *Earth Plan. Sci. Lett.* **408**, 48–56 (2014).

Them, T.R. II, Gill, B.C., Caruthers, A.H., Gröcke, D.R., Tulsky, E.T., Martindale, R.C., Poulton, T.P. & Smith, P.L*.* High-resolution carbon isotope records of the Toarcian Oceanic Anoxic Event (Early Jurassic) from North America and implications for the global drivers of the Toarcian carbon cycle. *Earth Plan. Sci. Lett.* doi: 10.1016/j.epsl.2016.11.021.

SI Figure 1.

SI Figure 2.

SI Figure 3.

SI Figure 4.
